# Effects of Exogenous MDA Supplementation to Diet on Antioxidant Capacity, Immunity and Body Color of Channel Catfish (*Ictalurus punctatus*)

**DOI:** 10.3390/vetsci13070632

**Published:** 2026-06-29

**Authors:** Li Li, Lu Zhang, Chunyu Xue, Leimin Zhang, Dongyu Huang, Mingchun Ren, Haifeng Mi, Hualiang Liang

**Affiliations:** 1 College of Fisheries and Life Science, Dalian Ocean University, Dalian 116023, China; 2 Tongwei Agricultural Development Co., Ltd., Key Laboratory of Aquatic Nutrition and Smart Farming, Ministry of Agriculture and Rural Affairs, Aquatic Health and Intelligent Aquaculture Key Laboratory of Sichuan Province, Chengdu 610093, China; 3 Wuxi Fisheries College, Nanjing Agricultural University, Wuxi 214081, China; 4 Key Laboratory of Integrated Rice-Fish Farming Ecology, Ministry of Agriculture and Rural Affairs, Freshwater Fisheries Research Center, Chinese Academy of Fishery Sciences, Wuxi 214081, China

**Keywords:** channel catfish, malondialdehyde, oxidative stress, immunity, body color

## Abstract

Malondialdehyde (MDA) is a common oxidation product in deteriorated aquatic feed, which easily induces oxidative damage and quality deterioration in farmed fish, yet its systemic effects on the physiological function and body color of channel catfish (*Ictalurus punctatus*) remain poorly understood. This study aimed to investigate the influences of dietary exogenous MDA on antioxidant capacity, immune function and body coloration of channel catfish. A 30-day feeding trial was carried out with four dietary MDA levels of 0, 22.3, 44.6 and 66.8 mg/kg. Relevant physiological and pigment indices of skin and muscle were determined. Exogenous MDA significantly increased plasma transaminase levels and tissue MDA content, decreased antioxidant enzyme activities, and altered the transcription of antioxidant and immune-related genes in skin and muscle, causing oxidative stress and immune dysfunction. It also suppressed tyrosinase activity, downregulated melanin-synthesis-related genes, reduced melanin deposition and promoted uranidin accumulation in the two tissues. Collectively, dietary exogenous MDA impairs antioxidant and immune performance, disrupts pigment metabolism, and ultimately leads to body yellowing in channel catfish.

## Simple Summary

Malondialdehyde (MDA) generated from oxidized feed threatens the health and product quality of farmed fish, while its specific harms to channel catfish (*Ictalurus punctatus*) remain unclear. This study investigated the effects of dietary exogenous MDA on the antioxidant capacity, immunity and body color of channel catfish via a 30-day feeding trial. The results demonstrated that exogenous MDA induced oxidative stress, decreased antioxidant enzyme activities and altered immune gene expression in skin and muscle, which impaired fish antioxidant and immune functions. It also inhibited tyrosinase activity, suppressed melanin synthesis and increased uranidin accumulation in the two tissues, resulting in abnormal body yellowing. These findings revealed MDA’s toxic mechanism and provided effective guidance for aquaculture feed quality control and improvement of catfish commercial value.

## 1. Introduction

In intensive aquaculture systems, oxidative stress is a critical determinant affecting fish health and production performance. During fish farming, multiple factors such as high-density rearing, environmental fluctuations, and dietary nutritional imbalance can induce excessive accumulation of ROS (reactive oxygen species) in fish organisms [1]. The imbalance between ROS generation and antioxidant neutralization engenders oxidative stress in fish [2]. It not only inflicts oxidative damage on key biomolecules, including lipids, proteins and DNA (deoxyribonucleic acid), but also activates inflammatory responses and disrupts normal physiological metabolism, ultimately affecting fish growth, immune function and product quality [3]. As the primary source of food and energy for farmed fish, feed runs the entire aquaculture process, and its quality is directly related to the redox homeostasis of fish. However, during transportation and storage, especially under high-temperature and high-humidity conditions, lipids in feed are prone to oxidative rancidity, generating aldehydes, ketones, alcohols and other potentially toxic oxidation derivatives [4,5]. Besides reducing the nutritive value of feed, these oxidation products may also induce or aggravate oxidative stress after being ingested by fish, thereby impairing growth performance, weakening immune competence and even deteriorating fish quality [6]. The implications of feed-derived lipid oxidation for fish growth and immune function have been extensively documented. For example, oxidized feed constituents have been demonstrated to compromise the antioxidant capacity of rainbow trout (*Oncorhynchus mykiss*) and to suppress growth performance and feed efficiency in *Rhynchocypris lagowskii* [7,8]. Here, the term antioxidant capacity refers to the levels of enzymatic antioxidants (SOD, CAT, GPx) and non-enzymatic antioxidant substances in fish tissues, rather than the lipid antioxidant capacity of fish muscle.

Earlier research has documented that oxidative stress experienced by fish during aquaculture can affect body coloration [9], and pigment deposition is directly correlated with oxidative stress levels [10]. Malondialdehyde (MDA) is widely recognized as both a major product of lipid peroxidation and a robust biomarker of oxidative injury in living organisms [11]. As a highly reactive aldehyde, MDA can induce cross-linking and structural alterations in key biomolecules, including proteins and nucleic acids, disrupting cellular structure and function, while concurrently amplifying oxidative stress and inflammatory cascades [12]. Consequently, establishing an oxidative stress model by supplementing exogenous MDA into diets has become a common method for simulating in vivo lipid peroxidation damage and exploring the pathogenic mechanism of oxidative stress under laboratory conditions [13].

Dietary malondialdehyde (MDA) is a common oxidation product in deteriorated aquatic feed that readily induces oxidative damage and quality deterioration in farmed fish. However, its systematic effects on the physiological function and body color of channel catfish (*Ictalurus punctatus*) remain poorly understood. Previous transcriptomic analysis has revealed that oxidative stress may be involved in body color abnormality by inducing inflammation and disrupting pigment metabolism [14]. On this basis, this study was designed to examine whether dietary exogenous MDA affects the antioxidant capacity, immune function, and body coloration of channel catfish. The findings are expected to provide a theoretical reference for feed quality control and offer practical guidance for improving the commercial value of farmed channel catfish.

## 2. Materials and Methods

### 2.1. Diet Preparation

MDA stock solution was prepared according to the current valid Chinese national mandatory food safety standard GB 5009.181-2016 [15] (National Food Safety Standard: Determination of malondialdehyde in food), which supersedes the obsolete GB/T 5009.181-2003. The specific preparation procedure was as follows: Accurately measure 31.5 mL of 1,1,3,3-tetraethoxypropane (Sigma-Aldrich, St. Louis, MO, USA, purity ≥ 99%), dissolve it in 95% ethanol and dilute to a final volume of 100 mL. The solution was stirred for 15 min to achieve full homogenization, and the concentration of the prepared MDA stock solution was determined as 1546.7 μmol MDA/mL via the TBA method with assay kits purchased from Jiancheng Bioengineering Institute (Nanjing, China) (kit number A003-1-2). All MDA solutions were prepared fresh for immediate use.

A total of four experimental groups were established, comprising one control group (Con) and three treatment groups (MDA1, MDA2, MDA3). The basal diet used in this experiment was commercial feed (control diet, Con). Volumes of 0.2, 0.4 and 0.6 mL of the prepared MDA solutions were taken and diluted to 100 mL, then sprayed evenly onto the surface of 1 kg of basal diet in each of the three groups, which were named MDA1, MDA2 and MDA3 groups in sequence. The MDA content in the diets was 22.3, 44.6 and 66.8 mg/kg, respectively. All MDA-supplemented diets were freshly prepared immediately before use, sprayed onto the basal formulation rapidly and uniformly, and the fish received the prepared diets right after compounding. The basal diet’s key ingredients and nutritional composition are outlined in Table 1.

### 2.2. Laboratory Fish and Husbandry

This 30-day trial was designed as a short-term subacute toxicological assay focusing on early biochemical, histological and transcriptomic toxic responses induced by MDA; therefore, routine sampling of growth performance indicators (weight gain, SGR, FCR, feed intake) was not arranged in the experimental protocol. Healthy channel catfish of uniform size and color were selected as experimental fish. A two-week acclimation period on a control diet was used to help the fish adapt to the rearing environment. The feeding experiment was conducted in net cages (2 m × 2 m × 2 m) at the Nanquan Experimental Base (Freshwater Fisheries Research Centre, Chinese Academy of Fishery Sciences). In this study, 180 channel catfish weighing 830 ± 5 g were randomly assigned to four experimental groups. Each group included three replicate net cages with 15 individuals stocked per cage, for a total of 12 cages. Over a 30-day feeding trial, four test diets were administered separately to each group. Fish were fed twice daily at 7:00 and 17:00, with equal rations provided at each feeding. The initial feeding rate was set at 3% of body weight and adjusted to weather conditions. Fish activity and health status were observed daily throughout the experiment, and feed consumption was documented regularly. During the entire experimental duration, aquaculture conditions were standardized as follows: dissolved oxygen concentration maintained above 6 mg/L, water temperature 24–29 °C, ammonia nitrogen concentration capped at 0.02 mg/L, and natural photoperiodic cycles. These parameters were monitored at regular intervals.

### 2.3. Sample Collection and Chroma Value Analysis

Sampling was conducted upon completion of the rearing trial, following a 24 h fasting period prior to sampling. From each cage, three healthy fish were chosen at random for tissue sampling and the determination of biochemical parameters. Anesthesia was induced in the selected test fish using 200 mg/L MS-222. Once the fish had become sedated, the color parameters of the dorsal skin and muscle tissue were measured. As described by Yu et al. [16], chromatic parameters including L* (lightness), a* (redness), and b* (yellowness) were recorded from the skin and muscle surfaces with a CR400 color difference meter. Blood was collected using sterile syringes, and plasma was harvested by centrifuging at 4000 rpm for 10 min under refrigerated conditions (4 °C). The fish were then dissected, and the dorsal skin as well as the muscle tissues were collected and preserved properly. All specimens were stored in a −80 °C ultra-low temperature freezer pending further determination and analysis.

### 2.4. Experimental Determination Method

#### 2.4.1. Analysis of Plasma Transaminase Activities and Tissue Antioxidant Indices

Commercially available assay kits from Jiancheng Bioengineering Institute (Nanjing, China) were used to determine plasma levels of aspartate aminotransferase (AST) and alanine aminotransferase (ALT). The microplate method was employed, and the respective kit numbers were (C010-2-1) and (C009-2-1). For antioxidant characterization, skin and muscle tissues were subjected to biochemical analysis using commercial assay kits from the same manufacturer. Total antioxidant capacity (T-AOC) was assessed by the ABTS method (A015-2-1). Enzymatic antioxidant indices, including total superoxide dismutase (T-SOD, WST-1 method, A001-3-2), glutathione peroxidase (GPx, colorimetric method, A005-1-2), and catalase (CAT, ammonium molybdate method, A007-1-1), were determined accordingly. Oxidative damage was evaluated by measuring malondialdehyde (MDA) levels through the TBA method (A003-1-2). All procedures followed the manufacturer’s instructions for each kit.

#### 2.4.2. ELISA Analysis of Pigment-Related Indices in Skin and Muscle

Skin and muscle tissues were analyzed for uranidin, melanin, and tyrosinase using an ELISA kit (Shanghai Enzyme-Linked Biotechnology Co., Ltd., Shanghai, China). The method is as follows: (1) Uranidin was quantified via a competitive assay (YJ270190). The method is briefly described as follows: purified uranidin antibodies are coated onto a microplate to form a solid-phase antibody. Uranidin samples are added to compete for binding with horseradish peroxidase (HRP)-labelled uranidin antigens. After washing, the substrate tetramethyl benzamine (TMB) is added to produce a color. The intensity of the color produced by the sample is inversely proportional to the uranidin content. Uranidin concentration was quantified spectrophotometrically at 450 nm using a microplate reader, with calibration against a constructed standard curve. (2) The competitive method (YJ234125) was utilized for melanin quantification in the tested sample. A brief description follows: The sample, standard, and HRP-labelled detection antibody are added in sequence to a melanin antibody-immobilized microplate. Following incubation and a wash step, TMB substrate is added and develops a blue color under peroxidase catalysis, which turns yellow after acid termination. The color intensity (measured at 450 nm) is positively correlated with melanin concentration and used to calculate its content. (3) Tyrosinase was quantified by competitive assay (H310-1-2). First, biotin-labelled antigen was added to a microplate pre-coated with antibody, and after a 30 min incubation at 37 °C, immune complexes were formed. Subsequently, unbound biotinylated antigen was eliminated by extensively washing the plate with PBST (phosphate-buffered saline containing Tween 20). Streptavidin–HRP was subsequently introduced and allowed to react at 37 °C for a further 30 min. Following washing, the bound HRP initiated TMB coloration, which changes from blue to yellow after acid termination. The OD was measured at 450 nm, and a negative association existed between this value and sample antigen levels. All analytical procedures and calculations were conducted in accordance with the respective kit manuals.

#### 2.4.3. RT-qPCR Analysis

Skin and muscle tissues were processed for total RNA extraction using the RNAiso Plus Kit (Vazyme, Nanjing, China). RNA concentration and quality were evaluated on a NanoDrop 2000 spectrophotometer (Thermo Fisher Scientific, Wilmington, DE, USA), with the quality standard set at an A260/280 ratio of between 1.8 and 2.0. Quantitative RT-PCR was conducted with the One Step SYBR Green Kit (Vazyme, Nanjing, China) on a CFX96 Touch instrument (Bio-Rad, Hercules, CA, USA). In this one-pot system, reverse transcription and quantitative amplification reactions were completed simultaneously within a single reaction tube using total RNA as the direct template, without separate cDNA synthesis. Given the well-documented stable expression of the glyceraldehyde-3-phosphate dehydrogenase (*gapdh*) gene across multiple tissues in relevant channel catfish studies, it was selected as the internal control [10,17]. Primer sequences, primer amplification efficiencies and corresponding *R*^2^ values for all target and reference genes are summarized in Table 2. Standard curves were generated via serial gradient dilutions of pooled total RNA extracted with the Vazyme RNA kit (seven concentration gradients, three technical replicates per gradient) to calculate amplification efficiency in accordance with MIQE guidelines. Primers were synthesized by Shenggong Bioengineering (Shanghai) Co., Ltd. (Shanghai, China). The fold differences in transcript levels between experimental cohorts were mathematically determined according to the 2^−ΔΔCt^ outlined by Schmittgen et al. [18].

### 2.5. Data Analysis

All statistical analyses were performed using SPSS 26.0. Prior to statistical analysis, the Shapiro–Wilk test was applied to assess data normality, and Levene’s test was used to verify homogeneity of variance. One-way ANOVA with Tukey’s post hoc test was subsequently applied to datasets meeting both assumptions for group comparisons. Results are reported in the format of means ± SD, and differences with *p* < 0.05 were considered statistically significant. Graphical visualization was conducted with GraphPad Prism 8.0.

## 3. Results

### 3.1. Effects of Exogenous MDA on Plasma Transaminase Activities of Channel Catfish

**Figure 1:**
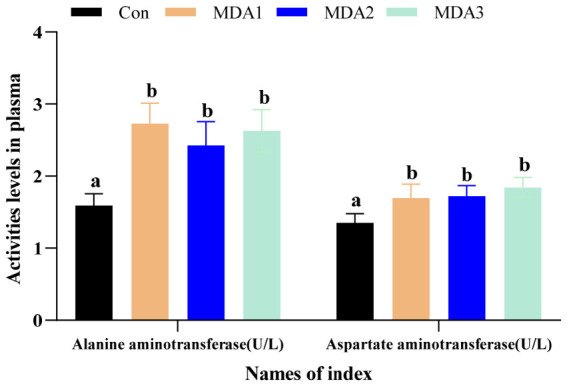
The levels of AST and ALT in plasma. Results were expressed in the format of means ± SD. Significant disparities between treatments were denoted by differing superscripts (*p* < 0.05).

Figure 1 showed that plasma samples from MDA-treated channel catfish exhibited significant upregulation of both transaminase indices, specifically AST and ALT, when compared with the untreated cohort (*p* < 0.05). AST measurements peaked at the highest values in the MDA3 group, whereas ALT activity attained its peak within the MDA1 treatment arm (*p* < 0.05).

### 3.2. Effects of Exogenous MDA on Antioxidant Capacity in Skin and Muscle of Channel Catfish

**Figure 2:**
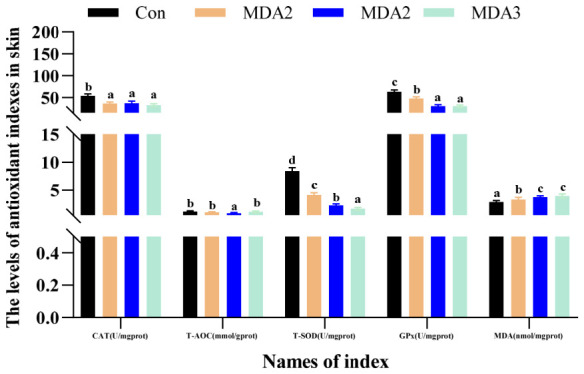

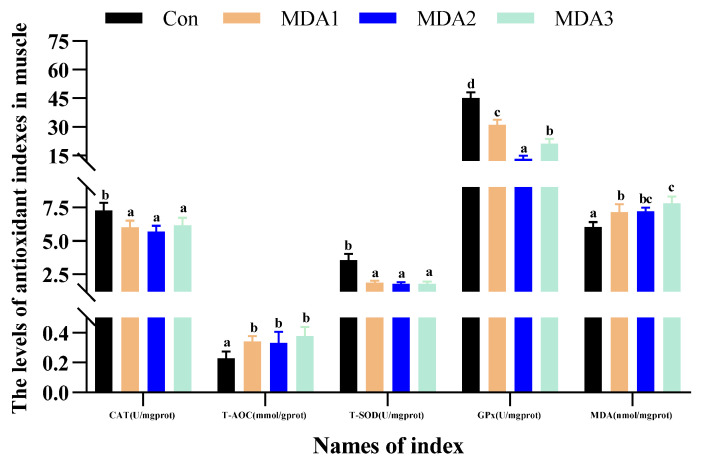
Antioxidant capacity indices in skin and muscle. Results were expressed in the format of means ± SD. Significant disparities between treatments were denoted by differing superscripts (*p* < 0.05).

Figure 2 shows that, relative to the control, skin from MDA-treated fish exhibited significantly compromised CAT, T-SOD and GPx profiles alongside elevated MDA burdens (*p* < 0.05), with T-AOC additionally suppressed in the MDA2 group (*p* < 0.05). In muscle, the MDA1-MDA3 groups showed equivalent enzymatic reductions, whereas both MDA contents and T-AOC levels were markedly elevated (*p* < 0.05).

### 3.3. Effects of Exogenous MDA on Antioxidant and Immune-Related Gene Expression in Skin and Muscle of Channel Catfish

**Figure 3:**
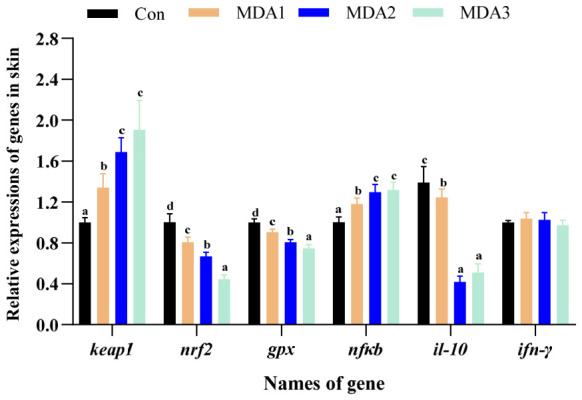

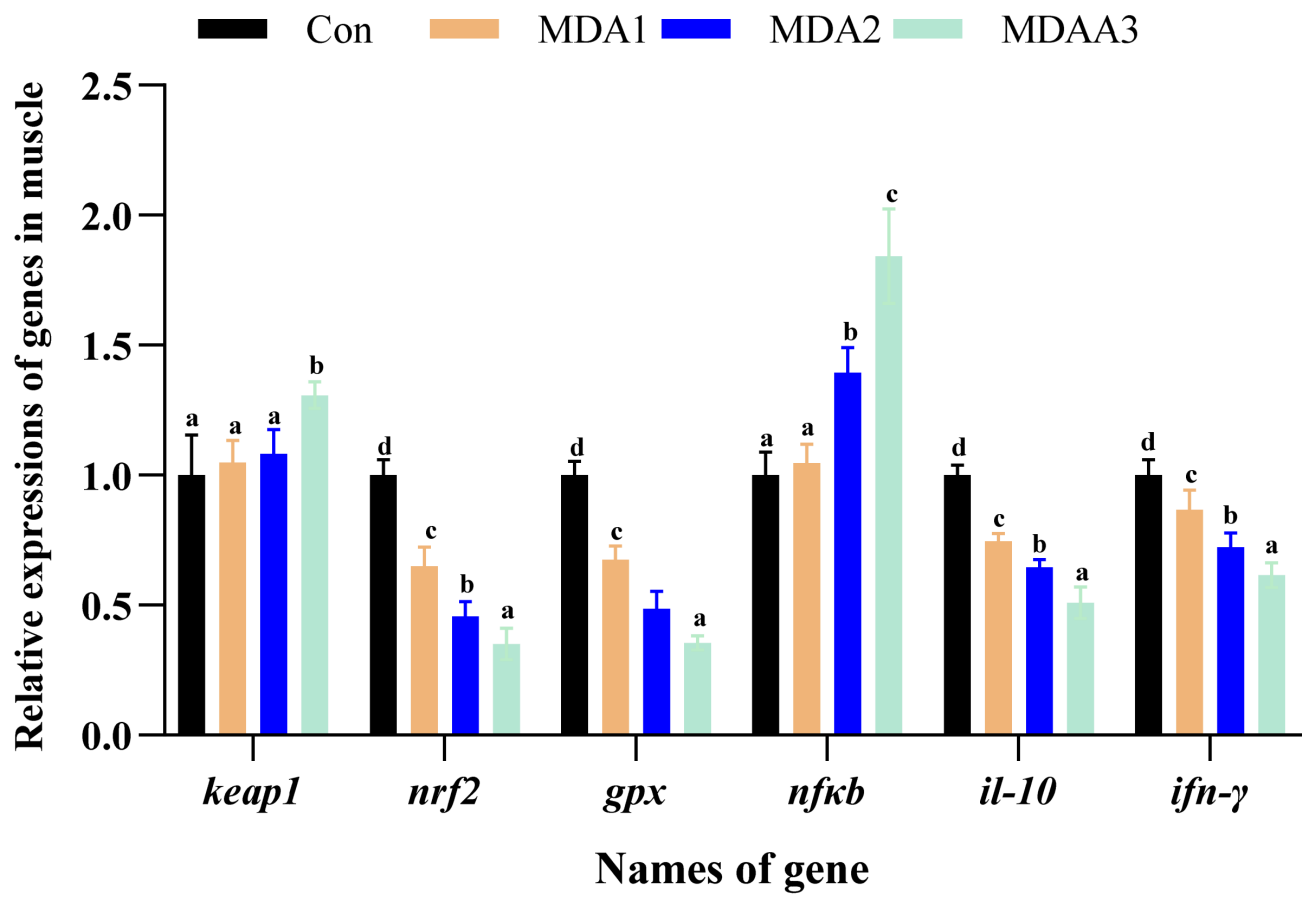
The expression levels of genes governing oxidative defense and immune function in skin and muscle. Results were expressed in the format of means ± SD. Significant disparities between treatments were denoted by differing superscripts (*p* < 0.05); bars lacking superscripts indicate no significant intergroup differences (*p* > 0.05).

Figure 3 shows that, relative to the control, MDA-treated fish skin exhibited significant downregulation of *nrf2*, *gpx* and *il-10* transcripts, whereas *keap1* and *nfκb* were markedly upregulated (*p* < 0.05). The mRNA levels of *ifn-γ* were unaffected by MDA treatment (*p* > 0.05). In muscle, treatment with the MDA1-MDA3 groups significantly reduced the mRNA levels of *ifn-γ*, *nrf2*, *gpx* and *il-10* (*p* < 0.05). MDA3 group markedly increased *keap1* gene expression, while the MDA2 and MDA3 groups significantly increased *nfκb* gene expression (*p* < 0.05).

### 3.4. Effects of Exogenous MDA on Skin and Muscle Coloration and Pigment Deposition of Channel Catfish

**Figure 4:**
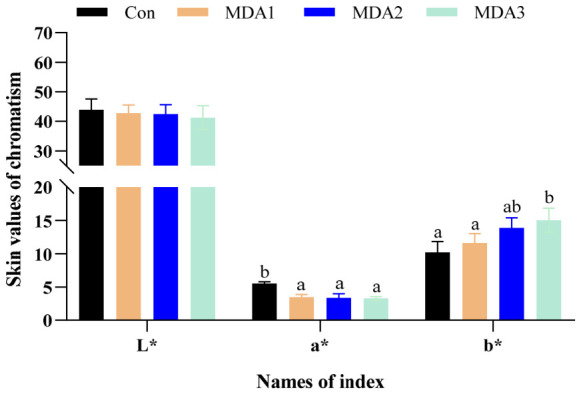

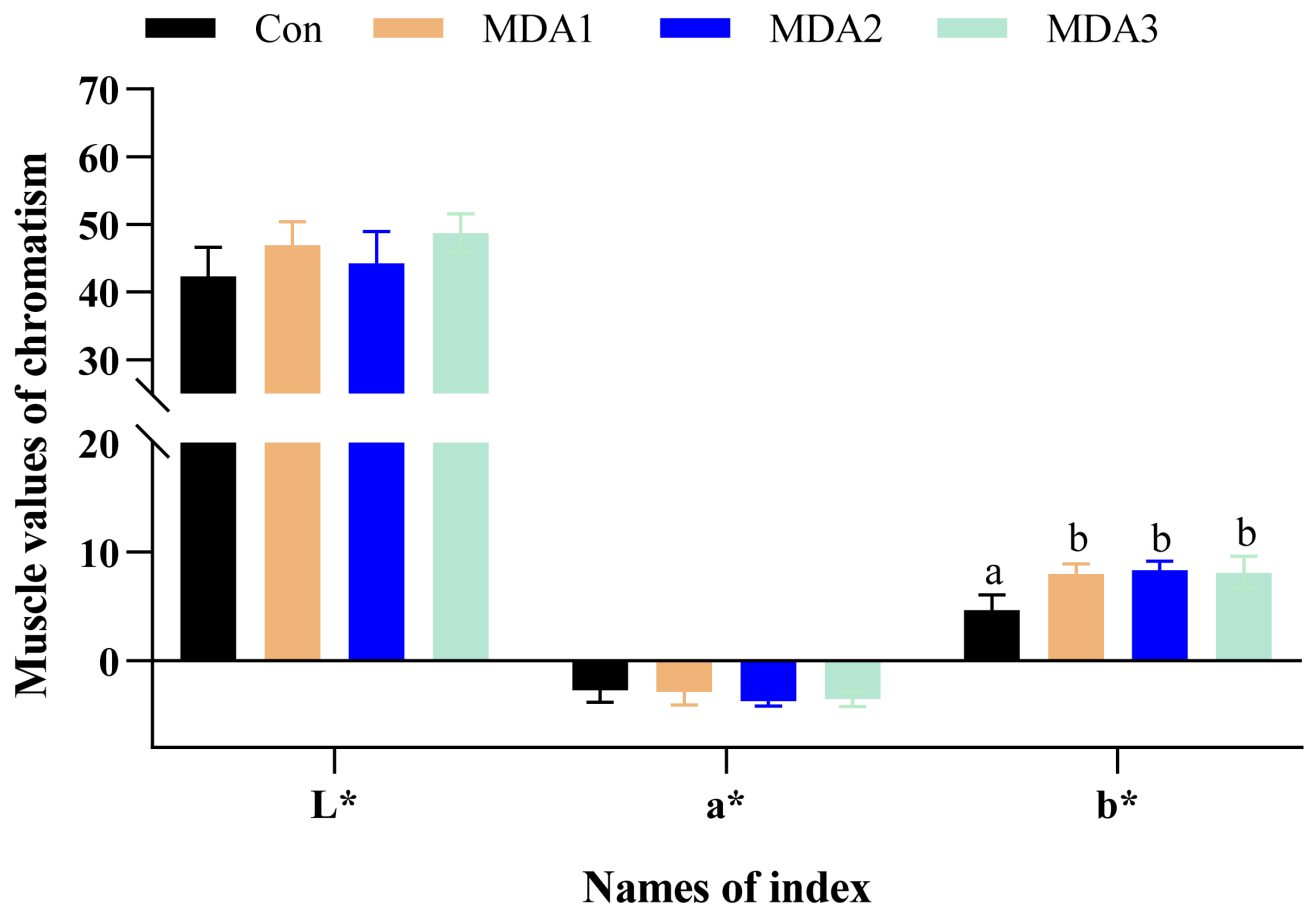
The chromaticity values of skin and muscle. Results were expressed in the format of means ± SD. Significant disparities between treatments were denoted by differing superscripts (*p* < 0.05); bars lacking superscripts indicate no significant intergroup differences (*p* > 0.05).

Figure 4 shows that, relative to the control, the L* value of the skin in the MDA1, MDA2 and MDA3 groups showed a gradual decrease as MDA levels increased, whilst the a* value decreased significantly (*p* < 0.05) and the b* value gradually increased; in the MDA3 group, the b* value increased significantly (*p* < 0.05). In muscle, the value of L* and a* showed no significant changes among groups (*p* > 0.05), whereas the value of b* was markedly increased in the MDA1-MDA3 groups (*p* < 0.05).

**Figure 5:**
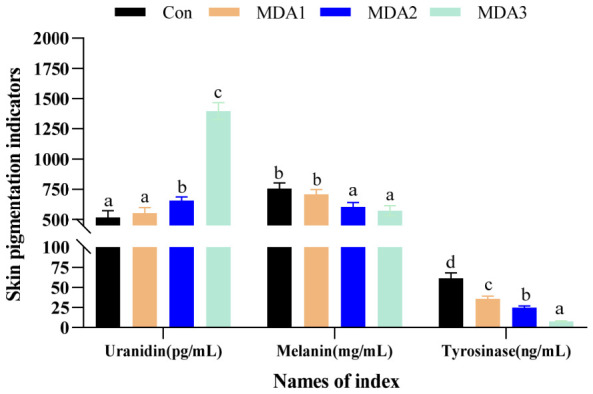

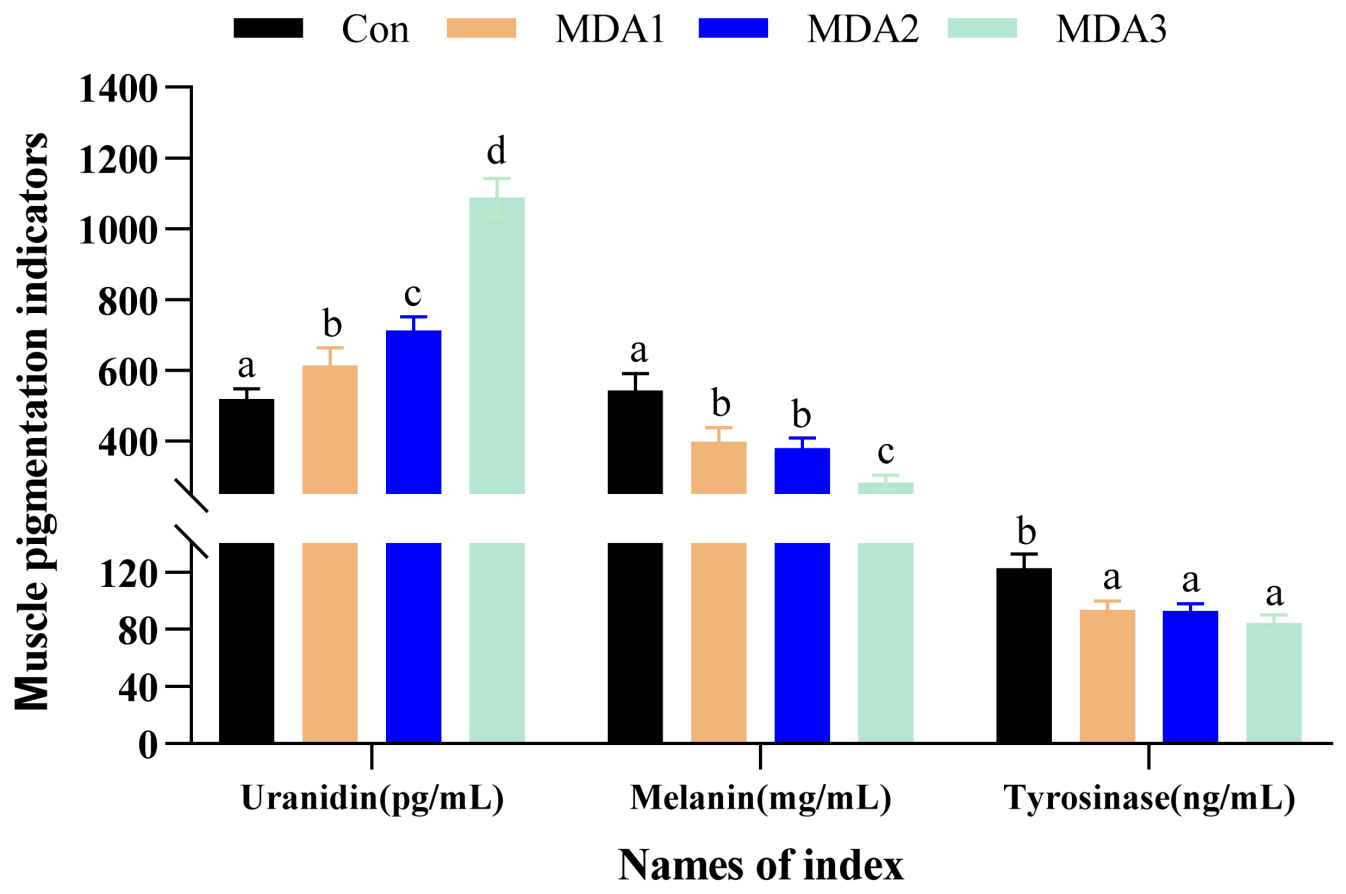
The levels of tyrosinase abundance and pigmentation in skin and muscle of channel catfish. Results were expressed in the format of means ± SD. Significant disparities between treatments were denoted by differing superscripts (*p* < 0.05).

As shown in Figure 5, uranidin content in skin and muscle increased with increased MDA level. Compared to controls, significant elevations occurred in skin (MDA2 and MDA3) and in muscle (all MDA groups) (*p* < 0.05). An opposing pattern characterized melanin dynamics. The skin melanin content was significantly reduced under MDA2 and MDA3 groups (*p* < 0.05), whereas muscle melanin showed significant reduction across all MDA-treated groups (*p* < 0.05). All groups treated with MDA exhibited significantly lower tyrosinase activity in skin and muscle (*p* < 0.05), and the degree of inhibition increased with higher MDA concentrations, exhibiting a dose-dependent relationship.

### 3.5. Effects of Exogenous MDA on Pigment-Related Genes in Skin and Muscle of Channel Catfish

**Figure 6:**
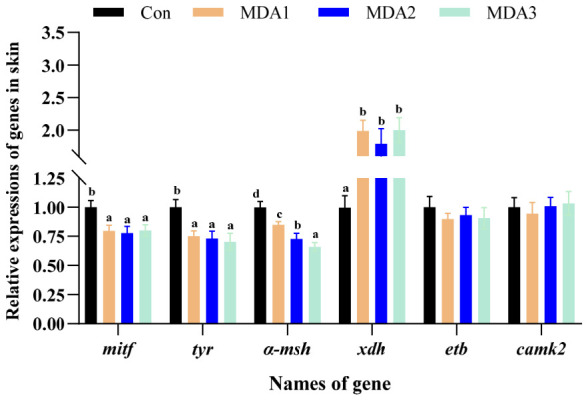

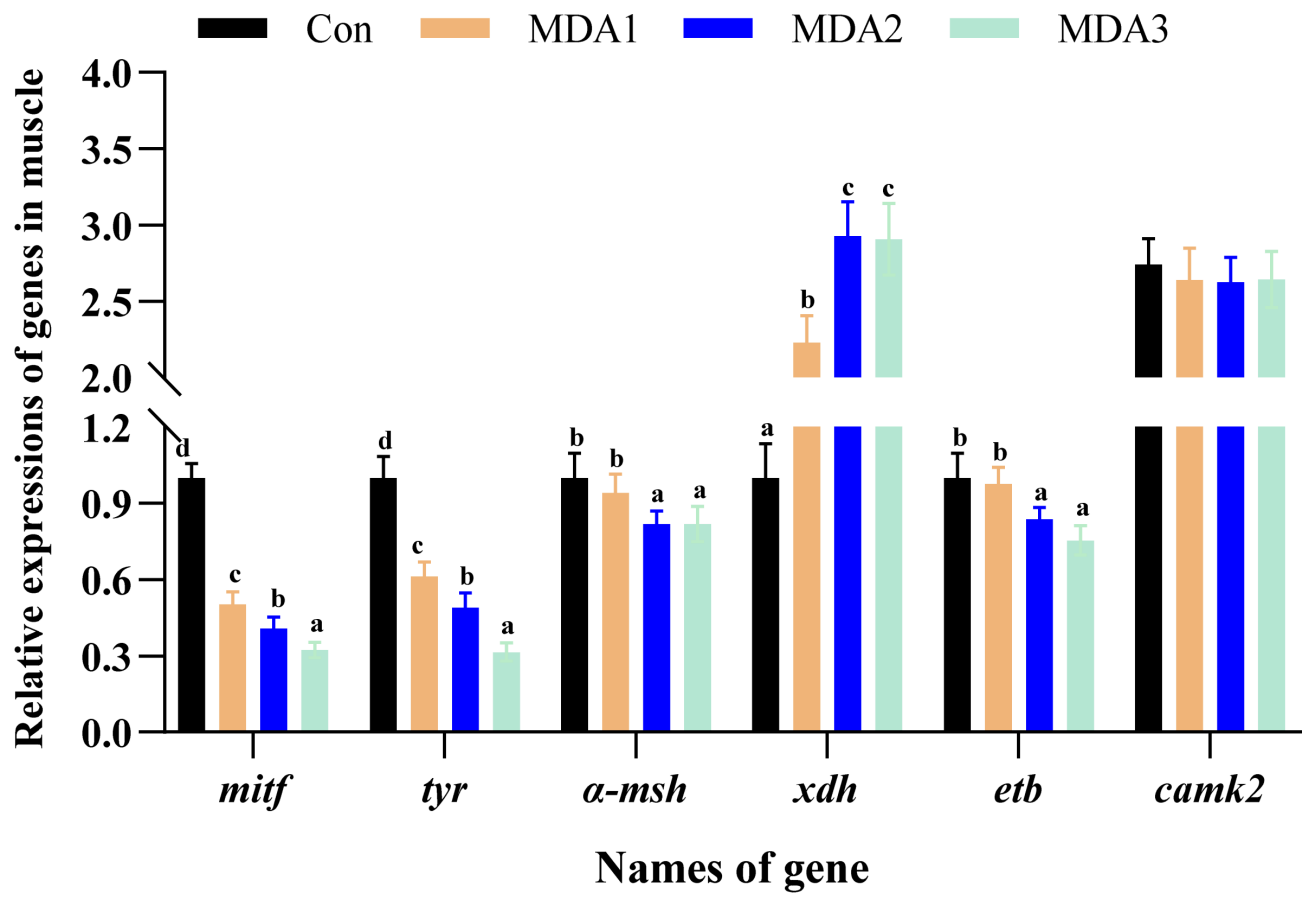
The mRNA levels of pigment synthesis-associated genes in skin and muscle. Results were expressed in the format of means ± SD. Significant disparities between treatments were denoted by differing superscripts (*p* < 0.05); bars lacking superscripts indicate no significant intergroup differences (*p* > 0.05).

Figure 6 shows that, in reference to the untreated cohort, the mRNA levels of *mitf*, *tyr* and *α-msh* in skin were significantly attenuated under MDA1-MDA3 groups, whereas the mRNA levels of *xdh* were markedly upregulated (*p* < 0.05). The mRNA levels of *etb* and *camk2* displayed no notable differences between groups (*p* > 0.05). In muscle, treatment with the MDA1-MDA3 groups resulted in reduced mRNA levels of the *mitf* and *tyr*, alongside a distinct upregulation of the *xdh* gene expression (*p* < 0.05). MDA2 and MDA3 groups significantly downregulated the expression of *α-msh* and *etb* (*p* < 0.05). However, no obvious statistical disparities in the mRNA levels of *camk2* were found across different treatment groups (*p* > 0.05).

## 4. Discussion

Our findings suggested that adding exogenous MDA to the diet induced oxidative stress in channel catfish. Plasma biochemical parameters reflect an overall physiological state of the organism, particularly liver health, which was the central organ for metabolic processes in the body, and plasma ALT and AST activities are important indicators for assessing the extent of hepatocyte damage [19]. When hepatocytes are damaged, large amounts of ALT and AST are released from the cells into the plasma, leading to significantly increased levels in plasma [20]. The present results showed that plasma activities of AST and ALT in MDA-treated groups were elevated relative to the control group, corroborating previous observations by Zhang et al. [21] and Shi et al. [17]. Based on these results, exogenous MDA induced oxidative stress and tissue damage in fish. Furthermore, at the tissue level, escalating exogenous MDA exposure elicited pronounced suppression of endogenous antioxidant enzyme activities, encompassing T-SOD, CAT, and GPx, concomitant with a marked surge in MDA accumulation as the terminal product of lipid peroxidation. This indicated that MDA induced oxidative stress in the organism, leading to impairment of the antioxidant defense system and lipid peroxidation damage. These findings aligned with an independent study demonstrating that exogenous MDA supplementation in the diet could inhibit the Keap1/Nrf2/ARE signaling pathway in hybrid groupers (*Epinephelus fuscoguttatus* ♀ × *E. lanceolatus* ♂), provoke oxidative stress, and consequently impair antioxidant defense [22]. Collectively, the findings from this section indicated that exogenous MDA disrupted the redox homeostasis and triggered marked oxidative stress in channel catfish [23]. To eliminate excessive intracellular ROS, fish have evolved an innate antioxidant defense system, encompassing antioxidant enzymes such as T-SOD, CAT, and GPx, alongside non-enzymatic antioxidants exemplified by GSH. Changes in the activities or contents of these indicators can reflect the physiological status and antioxidative capacity [24,25]. MDA constitutes a hallmark biomarker of lipid peroxidation, and its levels directly reflect the extent of this process and the associated oxidative damage in vivo [26]. Significant reductions in T-SOD, CAT, GPx and T-AOC indicate that under sustained ROS attack, the synthesis of antioxidant enzymes is inhibited, compensatory regulation is compromised, and overall antioxidant function is markedly impaired [27,28]. In Nile tilapia (*Oreochromis niloticus*), intraperitoneal injection of H_2_O_2_ to induce oxidative stress has been shown to significantly elevate plasma and hepatic MDA levels, while levels of CAT, T-SOD and GSH decrease [29]. Another investigation demonstrated that a diet enriched with oxidized fish oil significantly increased hepatic MDA content in channel catfish, while markedly reducing CAT, T-SOD, GPx activities and T-AOC levels [17]. Comparable outcomes, studies on Wuchang bream (*Megalobrama amblycephala*) [30] and tilapia (*Oreochromis niloticus*) [31] have also found that oxidized fish oil in feed significantly reduces antioxidant enzyme activity in these aquatic species and increases their plasma transaminase activity. Our findings showed that, in comparison to the control group, tissues from MDA-treated individuals presented significantly reduced activity of CAT, T-SOD and GPx in skin as well as muscle, alongside a sharp increase in endogenous MDA accumulation. Notably, the level of T-AOC in skin decreased significantly in MDA2 groups, whereas the level of T-AOC in muscle exhibited a compensatory elevation. As the primary physical barrier of the body against exogenous stressors, the skin contains antioxidant substances that are rapidly depleted, rendering the total antioxidant capacity unable to maintain homeostasis via compensatory regulation and consequently leading to MDA accumulation [32]. Consistent with both our findings and earlier reports, it can be concluded that channel catfish exposed to oxidative stress during aquaculture exhibit a compromised antioxidant system, reduced ROS scavenging capacity, and intensified lipid peroxidation. These alterations ultimately result in tissue oxidative damage and disruption of physiological functions in the organism.

In terms of antioxidant regulation, the Keap1/Nrf2/ARE axis represents the core pathway mediating the oxidative stress response in organisms [33]. In this pathway, *nrf2* serves as a key transcription factor that activates the transcription of downstream antioxidant genes, while *keap1* negatively regulates the antioxidant defense system by blocking *nrf2*’s nuclear import and promoting its degradation [34]. In this study, oxidative stress induced by MDA markedly upregulated *keap1* expression in the skin and muscle tissues, whereas it significantly suppressed *nrf2* and its downstream antioxidant gene, *gpx*. Moreover, such inhibitory effects were enhanced with the increase in MDA concentration, which corroborated previous reports. Shi et al. demonstrated that in the liver of channel catfish [17]. In addition, Yang et al. also confirmed that intake of oxidized fish oil remarkably increased intestinal ROS level and impaired the repair capacity of the antioxidant system in Wuchang bream [35]. These findings suggested that exogenous MDA induced oxidative stress, inhibited the Keap1/Nrf2/ARE signaling pathway, which in turn downregulated the transcription of antioxidant genes and weakened the organism’s ability to eliminate ROS. This further confirmed that the antioxidant defense system of fish was markedly inhibited under oxidative stress conditions. Organism health relies not only on a sound antioxidant defense system but is also critical for immune function. At the level of immune modulation, the NF-κB signaling pathway acts as a core modulator of fish inflammatory responses and the transcriptional activity of immune-associated genes [36]. Altered expression of *nfκb*, a key regulatory gene within this pathway, can affect the transcription of downstream cytokines with opposing inflammatory roles, including *ifn-γ* (pro-inflammatory) and *il-10* (anti-inflammatory). In this study, adding exogenous MDA markedly enhanced the transcription of *nfκb* in the skin and muscle tissues, with transcript levels increasing in parallel with adding levels of exogenous MDA, while also leading to a marked downregulation of the anti-inflammatory cytokine *il-10*. In the skin, *ifn-γ* expression first increased and then decreased, whereas in the muscle, *ifn-γ* expression was significantly suppressed. These findings revealed a close correlation between MDA-induced oxidative stress and inflammatory responses. As a central regulator of inflammatory responses, upregulated *nfκb* can activate the transcription of downstream pro-inflammatory cytokines and exacerbate systemic inflammatory reactions [37]. Playing a central role in resolving inflammation, *il-10* acts to restrain the release of pro-inflammatory factors, thereby preserving immunological homeostasis [38]. The expression imbalance between these two genes serves as an important molecular hallmark of inflammatory damage in fish. Earlier research has confirmed that oxidative stress promotes inflammation in fish via activation of the NF-κB pathway, whilst simultaneously suppressing the expression of the anti-inflammatory cytokine *il-10*. For instance, Zhang et al. confirmed that feeding *Rhynchocypris lagowskii* with oxidized fish oil led to high expression of pro-inflammatory cytokines accompanied by low expression of anti-inflammatory cytokines [39]. In addition, Zhang et al. also found that prolonged consumption of oxidized fish oil aggravated inflammation in juvenile swamp eel (*Monopterus albus*) via the upregulation of pro-inflammatory cytokines, which in turn led to compromised immune function [40]. This study revealed that the imbalance between *nfκb* and *il-10* induced by MDA treatment indicated that oxidative stress not only damages the antioxidant system of fish, but also triggers disorder of systemic inflammatory responses, further exacerbating tissue damage.

Oxidative stress could affect the body color and flesh color of fish, mainly manifested as dull and faded body color, as well as whitening, yellowing or loss of vividness in flesh color. Studies have shown that microplastic-induced oxidative stress triggers degradation of the blue structural color and reduced glossiness in the caudal fin of peacock fish (*Poecilia reticulata*), indicating oxidative stress disrupts iridocyte integrities [41]. Under oxidative stress, carotenoid levels in yellow catfish (*Pelteobagrus fulvidraco*) muscle decline, causing the flesh color to change from golden yellow to pale white [42]. Environmental factors such as hypoxia and low temperature trigger oxidative stress, which reduces the flesh b* and C* values of Nile tilapia, resulting in pale flesh color and loss of vividness [43]. This study found that as the MDA concentration increased in the treatment groups, the a* values of both skin and muscle in channel catfish decreased, while the overall skin b* values increased, deviating from the normal body color. Collectively, these results indicated that oxidative stress driven by MDA negatively affects the skin coloration of channel catfish under conditions of high ROS levels. Surplus ROS produced under oxidative conditions progressively build up and disrupt the signal transduction routes associated with melanin synthesis [44]. For instance, oxidized lipids affect the differentiation and development of fish melanocytes, reduce the population of mature melanocytes, thereby leading to inadequate melanin synthesis and indirectly elevating skin yellowness value [45]. Melanin is a biological pigment synthesized from tyrosine via a cascade of biochemical reactions, which is classified into eumelanin (black/brown) and pheomelanin (red/yellow). The content and distribution of melanin determine body skin coloration [46,47]. Tyrosinase acts as the key regulatory enzyme in the melanin biosynthetic route, and its catalytic action directly modulates the rate of melanin production [48,49]. Xanthophores are essential for fish body coloration and are capable of transdifferentiating with melanocytes [50]. Our findings indicated that increasing MDA supplementation resulted in a dose-dependent suppression of tyrosinase activity and melanin content in skin and muscle tissues. This correlation was consistent with the well-established conclusion that inhibiting tyrosinase activity can effectively reduce melanin production [51,52,53], which strongly demonstrated that the inhibition of tyrosinase activity and blockade of normal melanin synthesis represent one of the core mechanisms by which oxidative stress induces changes in body color and flesh color [54]. Meanwhile, oxidative stress promoted the manifestation of yellowness in the skin. The main uranidin in channel catfish’s skin and muscle are carotenoids such as lutein, zeaxanthin and isoflavone. The conjugated double bonds in their molecular structures endow them with antioxidant functions of scavenging free radicals and maintaining ROS homeostasis [55]. This study demonstrated that skin and muscle uranidin content increased markedly in parallel with increasing MDA concentration. It was speculated that this change might be attributed to the shunting of metabolic substrates to the uranidin deposition pathway following the inhibition of melanin synthesis. Alternatively, it might serve as an active protective response of the organism against oxidative damage through carotenoid accumulation [56]. The underlying mechanisms remain to be further investigated. Further combined with the analysis of gene expression levels, the molecular mechanism by which oxidative stress regulates abnormal body coloration in channel catfish can be further elucidated. The genes including *mitf*, *tyr*, and *α-msh* are involved in regulating melanin synthesis at three distinct levels: transcriptional, enzymatic, and hormonal [57]. To be specific, microphthalmia-associated transcription factor is encoded by *mitf*, tyrosinase by *tyr*, and α-melanocyte-stimulating hormone by *α-msh*. Both *etb* and *camk2* represent key regulatory components of the melanin synthesis signaling pathway. Abnormal expression levels or activities of the two genes can directly lead to abnormal body color formation in fish by affecting melanocyte differentiation, migration, and the transcriptional regulation of core genes responsible for melanin synthesis [58,59]. Our results indicated that MDA treatment markedly downregulated the transcript levels of *mitf*, *tyr* and *α-msh* in skin and muscle tissues, while *etb* and *camk2* levels also presented a decreasing trend. These results aligned with an earlier study conducted by Ye et al. [60], who found that administering oxidized fish oil to yellow catfish (*Pelteobagrus fulvidraco*) induced oxidative stress and significant downregulation of key melanin synthesis genes such as *tyr*, *mitf* and *etb*, with the effect being dose-dependent. In addition, a study of zebrafish (*Danio rerio*) has demonstrated that the loss of *etb* expression directly causes disorders in melanocyte differentiation and migration, eventually resulting in body color albinism and stripe disappearance [61]. Collectively, these results indicated that oxidative stress could block melanin production at the source by transcriptionally downregulating key synthesis genes. The homolog of the enzyme encoded by the *Xdh* gene is capable of synthesizing yellow-red pteridine pigments [62,63]. In this study, the transcript levels of the yellow pigment synthesis-related gene *xdh* increased significantly with escalating MDA doses, mirroring the dose-dependent rise in tissue uranidin content. In conclusion, exogenous MDA-induced oxidative stress mainly inhibits tyrosinase activity, disrupts the deposition balance between melanin and uranidin, and ultimately leads to skin yellowing in channel catfish.

This study has certain limitations. The 30-day short-term subacute MDA exposure trial only focused on early biochemical, histological and transcriptomic toxic responses, and growth-related indicators were not included in the experimental design, as obvious growth inhibition cannot be observed within a short exposure period. Long-term chronic feeding trials integrating growth and toxic injury detection will be conducted in subsequent research to systematically assess MDA toxicity, and multi-reference-gene normalization will be adopted in future qPCR analysis. Nevertheless, the core conclusions on early toxic mechanisms derived from the present data remain credible.

## 5. Conclusions

Exogenous MDA supplementation in the diet induced oxidative stress and impaired antioxidant capacity, as well as immune function, and caused oxidative tissue damage. By interfering with the pigment metabolic pathway, it inhibited melanin synthesis and promoted uranidin accumulation, ultimately leading to body color yellowing in channel catfish.

## Figures and Tables

**Table 1:** Composition of ingredients and nutrients in the basal diet (% dry basis).

Ingredients	Additional Level (%)	Ingredients	Additional Level (%)
Japanese fish meal ^a^	12.0	Fish oil	3.5
Soybean meal ^a^	30.0	Multi-dimensional multi-ore premixes ^b^	1.0
Rapeseed meal ^a^	14.0		
Wheat meal ^a^	15.5	L-lysine (70%)	0.3
High-lipid rice bran ^a^	15.5	Choline	0.2
Analyzed proximate composition			
Crude protein (%)		35.7	
Crude lipid (%)		8.4	
Grude ash (%)		15	
Moisture (%)		12.5	

**Note:** ^a^ Fish meal, soybean meal, rapeseed meal, wheat meal and high-lipid rice bran were obtained from Wuxi Tongwei feedstuffs Co., Ltd., Wuxi, China. ^b^ Multi-dimensional multi-ore premixes, including vitamin premixes and mineral premixes. Both were obtained from HANOVE Biotechnology Co., Ltd., Wuxi, China. Vitamin premixes (IU or mg/kg of diet): Vitamin A, 900,000 IU; Vitamin D, 250,000 IU; Vitamin E, 4500 mg; Vitamin K 3220 mg; Vitamin B1, 320 mg; Vitamin B2, 1090 mg; Vitamin B5, 2000 mg; Vitamin B6, 5000 mg; Vitamin B12, 116 mg; Pantothenate, 1000 mg; Folic acid, 165 mg; Biotin, 50 mg; Niacin acid, 2500 mg. Mineral premixes (g/kg of diet): calcium phosphate, 20 g; sodium chloride, 2.6 g; potassium chloride, 5 g; magnesium sulphate, 2 g; ferrous sulphate, 0.9 g; zinc sulphate, 0.06 g; cupric sulphate, 0.02 g; manganese sulphate, 0.03 g; sodium selenate, 0.02 g; cobalt chloride, 0.05 g; potassium iodide, 0.004 g.

**Table 2:** Primer sequences and gene abbreviations for RT-qPCR.

Genes	Primer Sequence	Primer Amplification Efficiency (%)	*R* ^2^	Primer Source
*mitf*	F: CCTGGACCATGTGGCAAGTT R: TGAACGTGTGTACAGGTCGG	98.5	>0.99	XM_017479247.3
*tyr*	F: GCAGTGAGACAAACAGAGAACG R: GCATGATGTTACGACGCACC	97.6	>0.99	XM_053677474.1
*α-msh*	F: TCAACCCTCTGGCCGAAATC R: GAAGGTAACCAGGCACACGA	101.3	>0.99	XM_015373818.1
*xdh*	F: CTCGCCACAACAACATGCAA R: GCTCTGACCAGGAGGGACTA	98.4	>0.99	XM_017475915.1
*etb*	F: CAGAGCAGTCTGGGTCAAGG R: ATGAGTACGTTTGGCCCGTT	99.3	>0.99	XM_017474261.1
*camk2*	F: AATCCAGCTCCACCGTTCAG R: TGATCTCCTGTTTGCGTGCT	101.8	>0.99	XM_017489404.1
*ifn-γ*	F: GGTTTGTAATCGAGCGGGGA R: TCCAAAGGTCACGCTGTACC	102.1	>0.99	NM_001200178.1
*keap1*	F: AGAGGTACGACCCGGAAAGA R: GCCGTCATAGCCACCCATTA	95.7	>0.99	XM_017482240.3
*nrf2*	F: GGCGTGGCAAGAACAAGGTAG R: TGAAGGGAGTAGTCGTTAGGG	98.8	>0.99	XM_017470076.3
*gpx*	F: TTTGTTTGTGCCGTGTTGAGT R: TGGGTGTAATCCCTGGTGGTC	96.7	>0.99	NM_001200741.1
*nfκb*	F: TGGCGCATCCTTTGCTTAGA R: AGACACAGCGGTGCATACAA	102.3	>0.99	XM_017490807.1
*il-10*	F: TGCAGGCTTACGAAAGGGTT R: CATGTCCAGCTCTCCCATGG	100.8	>0.99	XM_017450800.3
*gapdh*	F: ACCAATGAGAAGGCCTCTGC R: CATGTCCAGCTCTCCCATGG	96.6	>0.99	NM_001201199.1

**Note:** *mitf*, microphthalmia-associated transcription factor; *tyr*, tyrosinase; *α-msh*, α-melanocyte stimulating hormone; *xdh*, Xanthine dehydrogenase; *etb*, endothelin receptor type B; *camk2*, calcium/calmodulin-dependent protein kinase II; *ifn-γ*, interferon-γ; *keap1*, Kelch-like ECH-associated protein 1; *nrf2*, nuclear factor erythroid 2-related factor 2; *gpx*, glutathione peroxidase; *nfκb*, nuclear factor kappa-B; *il-10* = interleukin-10; *gapdh*, glyceraldehyde-3-phosphate dehydrogenase.

## Data Availability

The original contributions presented in this study are included in the article/Supplementary Materials. Further inquiries can be directed to the corresponding authors.

## Supplementary Materials

The following are available online at https://www.mdpi.com/article/10.3390/vetsci13070632/s1, Figure S1: Representative photographs of normal body skin color and dorsal muscle color in healthy channel catfish (*Ictalurus punctatus*), Figure S2: Representative photographs of body skin color and dorsal muscle color of yellowed channel catfish (*Ictalurus punctatus*).
