# Supplementary material for: Effects of Exogenous MDA Supplementation to Diet on Antioxidant Capacity, Immunity and Body Color of Channel Catfish (Ictalurus punctatus)
Source: Vet Sci. 2026 Jun 29;13(7):632. doi: 10.3390/vetsci13070632 (PMC13431366; doi:10.3390/vetsci13070632)
Supplement: Supplementary file 1 [file vetsci-13-00632-s001.zip › vetsci-4380120-supplementary.pdf]

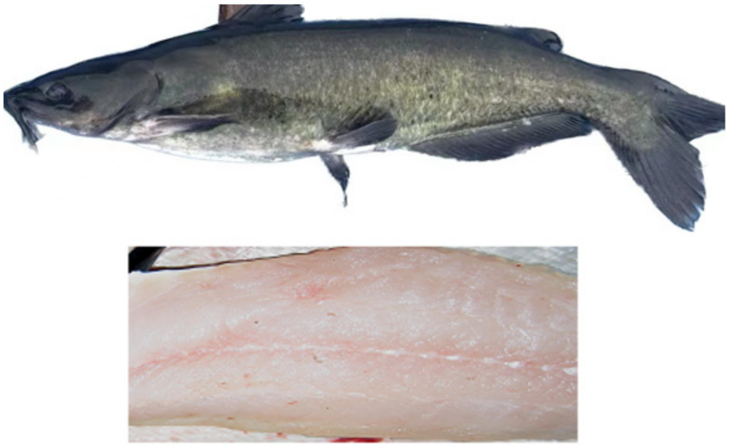

Figure S1: Representative photographs of normal body skin color and dorsal muscle color in healthy channel catfish (*Ictalurus punctatus*).

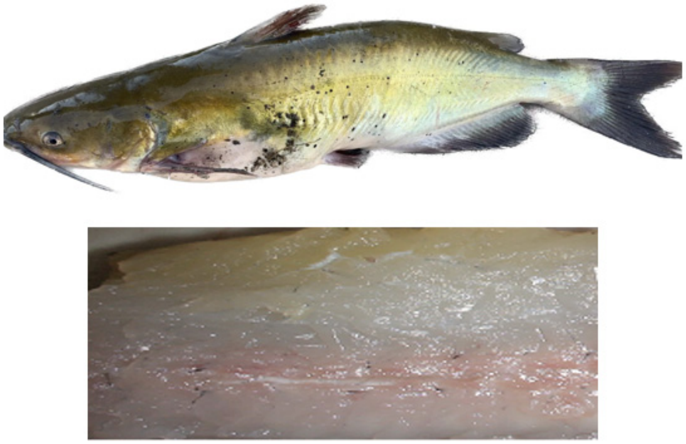

Figure S2: Representative photographs of body skin color and dorsal muscle color of yellowed channel catfish (*Ictalurus punctatus*).
